# Case Report: An unexpected diagnosis of cutaneous *Mycobacterium marinum* infection based on potato dextrose agar culture

**DOI:** 10.3389/fmed.2025.1709332

**Published:** 2025-11-14

**Authors:** Xiujiao Xia, Jiajia Li, Zehu Liu

**Affiliations:** Department of Dermatology, Hangzhou Third People’ s Hospital, Hangzhou Third Hospital Affiliated to Zhejiang Chinese Medical University, Hangzhou, China

**Keywords:** non-tuberculous mycobacteria, culture, potato dextrose agar, cutaneous infection, diagnosis

## Abstract

Non-tuberculous mycobacteria (NTM) are slender, non-motile, acid-fast bacilli widely distributed in diverse environmental reservoirs across the globe. In recent years, the incidence of NTM-related diseases has risen significantly, leading to their recognition as major emerging human pathogens. Cutaneous NTM infections typically arise following traumatic injury, surgical intervention, or cosmetic procedures. Due to their heterogeneous clinical presentations, these infections often pose diagnostic challenges. Löwenstein-Jensen medium and Middlebrook medium are widely employed in mycobacterial microbiology and are capable of supporting the growth of most NTM. However, their high cost and limited availability pose significant challenges for implementation in resource-limited settings (RLS). Previously, we successfully isolated a strain of *Mycobacterium marinum* from a clinical sample using potato dextrose agar (PDA) medium. This finding suggests that PDA may be applicable for the primary culture of NTM, particularly *M. marinum*. For RLS, this medium could represent a beneficial alternative to traditional mycobacterial culture media.

## 1 Introduction

Cutaneous infection caused by non-tuberculous mycobacteria (NTM), especially *Mycobacterium marinum* (a waterborne species), appear to be increasing in incidence (1). The diagnosis of NTM cutaneous infection requires the detection of acid-fast bacilli by microscopy, culture, and/or PCR on skin fluid or tissue biopsy specimens (2). Early identification is crucial, as many empirical antibiotic regimens are ineffective against NTM disease (3). However, the diagnosis of NTM cutaneous infections presents a considerable challenge for resource-limited settings (RLS).

Löwenstein-Jensen medium (LJ) and Middlebrook medium are traditionally employed for the primary isolation of mycobacteria in clinical microbiology (4–6). Prior to June 2021, we had not yet implemented the standardized operational procedure for mycobacterial culture. During this period, *M. marinum* was unexpectedly isolated on a potato dextrose agar (PDA) plate. We present it here as a practical reference for RLS.

## 2 Case description

**FIGURE 1:**
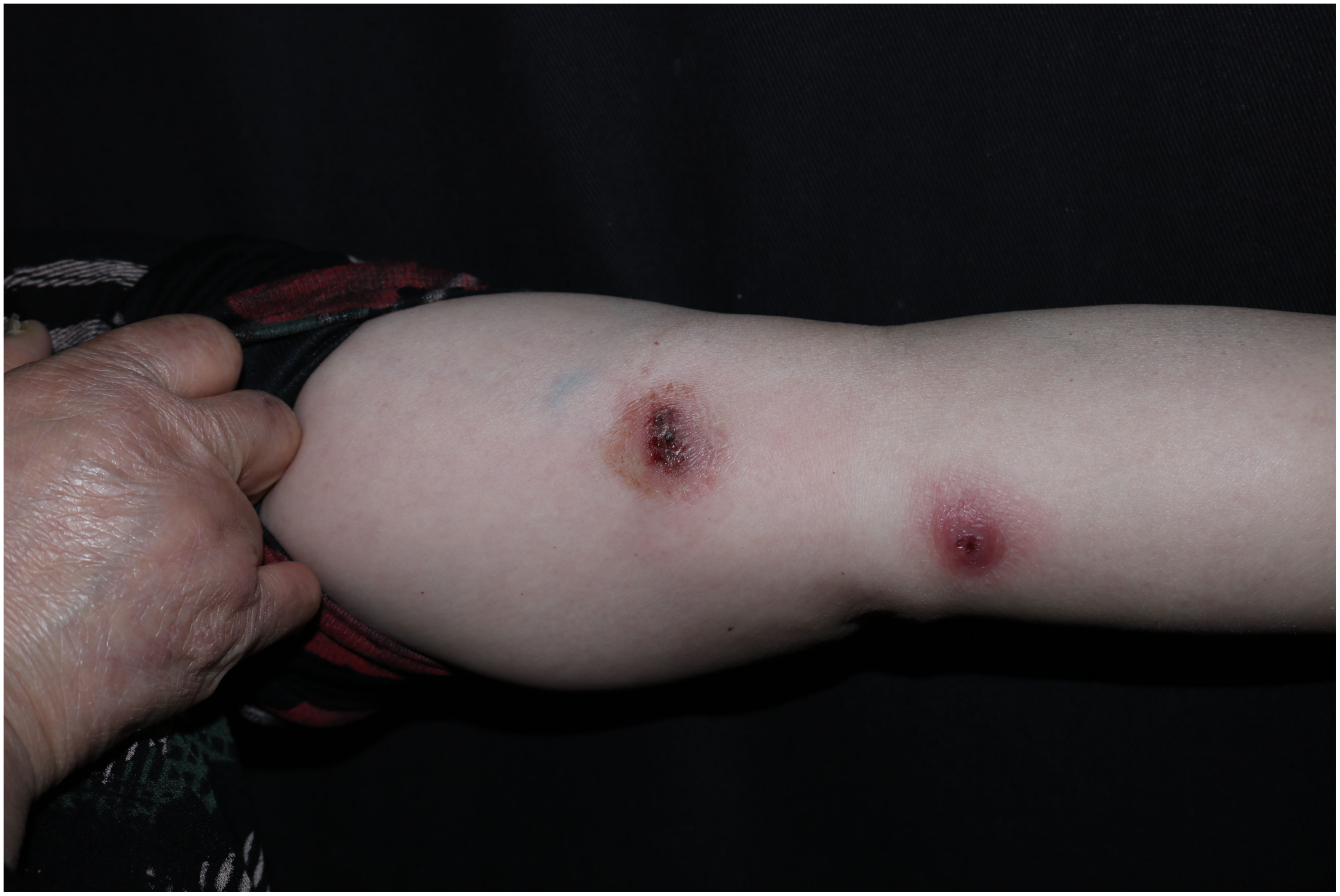
Two isolated erythematous nodules with superficial ulceration on the medial aspect of the left upper arm.

**FIGURE 2:**
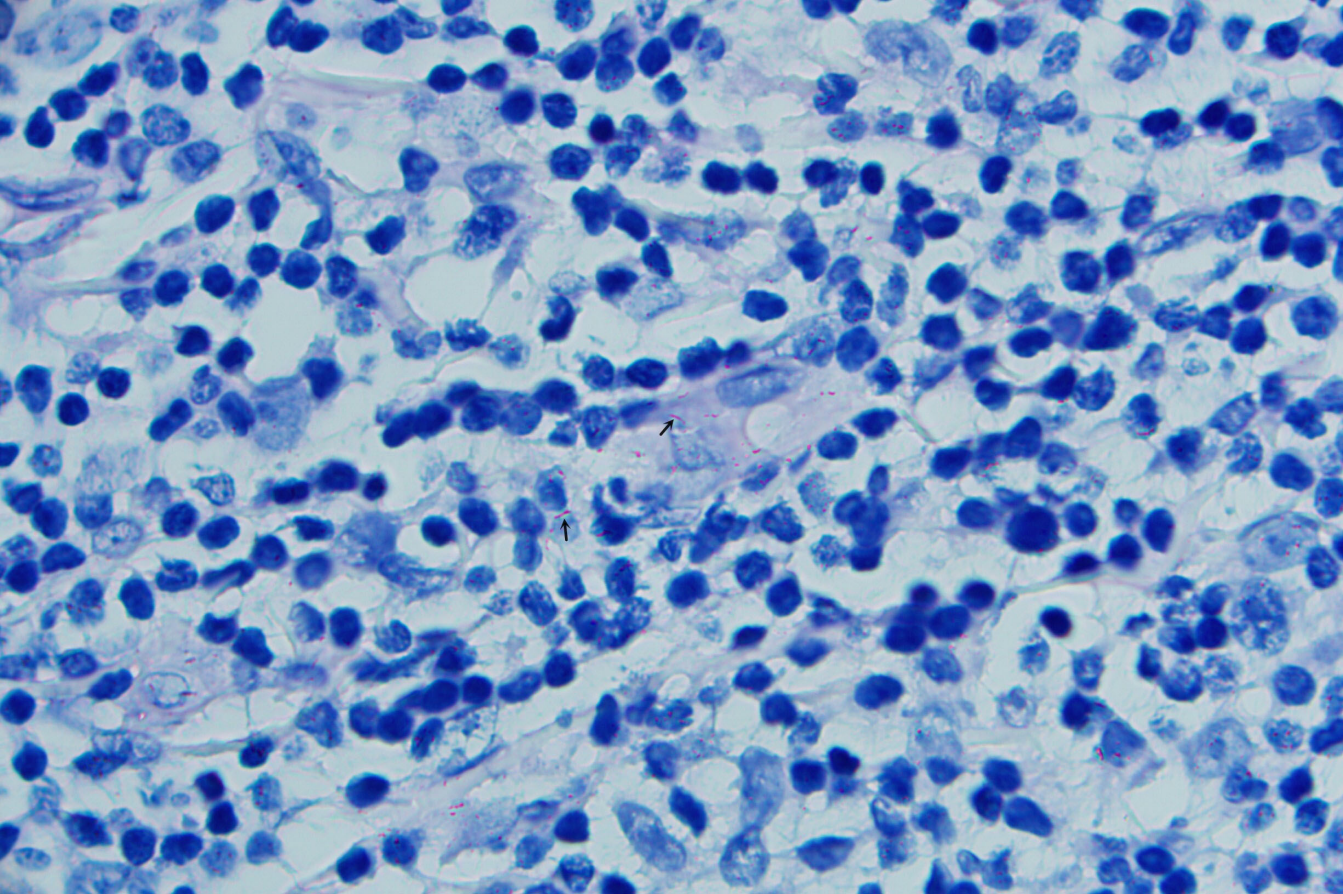
Histologic section showing short rod-shaped bacilli (acid-fast staining ×1000) (arrows).

A 52-years-old woman presented on April 28, 2020, with erythematous lesions on her left upper arm that had progressively enlarged, ulcerated, and persisted for 2 months. The patient worked in the aquatic trade and reported sustaining an accidental puncture wound to her left middle finger from a shrimp, which caused localized erythema and swelling. One month later, she developed pruritic nodules on her upper arm. After seeking care at a local hospital, she was diagnosed with sporotrichosis and treated initially with itraconazole (200 mg/day), followed by 10% potassium iodide solution (30 mL/day). However, her symptoms showed no improvement. Dermatological examination revealed two isolated erythematous nodules with superficial ulceration on the medial aspect of the left upper arm (Figure 1), accompanied by fragmented and thickened bilateral thumbnails. Histopathological analysis demonstrated hyperkeratosis with parakeratosis and serocrust deposition in the stratum corneum; Focal neutrophilic aggregates and pseudoepitheliomatous hyperplasia in the epidermis; Dense lymphohistiocytic infiltrates with scattered epithelioid cells, multinucleated giant cells, and neutrophilic sheets in the superficial-to-mid dermis; Perivascular lymphocytic predominance with fibroblastic proliferation and collagen deposition in the deep dermis. Acid-fast staining identified short rod-shaped bacilli (Figure 2). Regular laboratory investigations were otherwise unremarkable.

**FIGURE 3:**
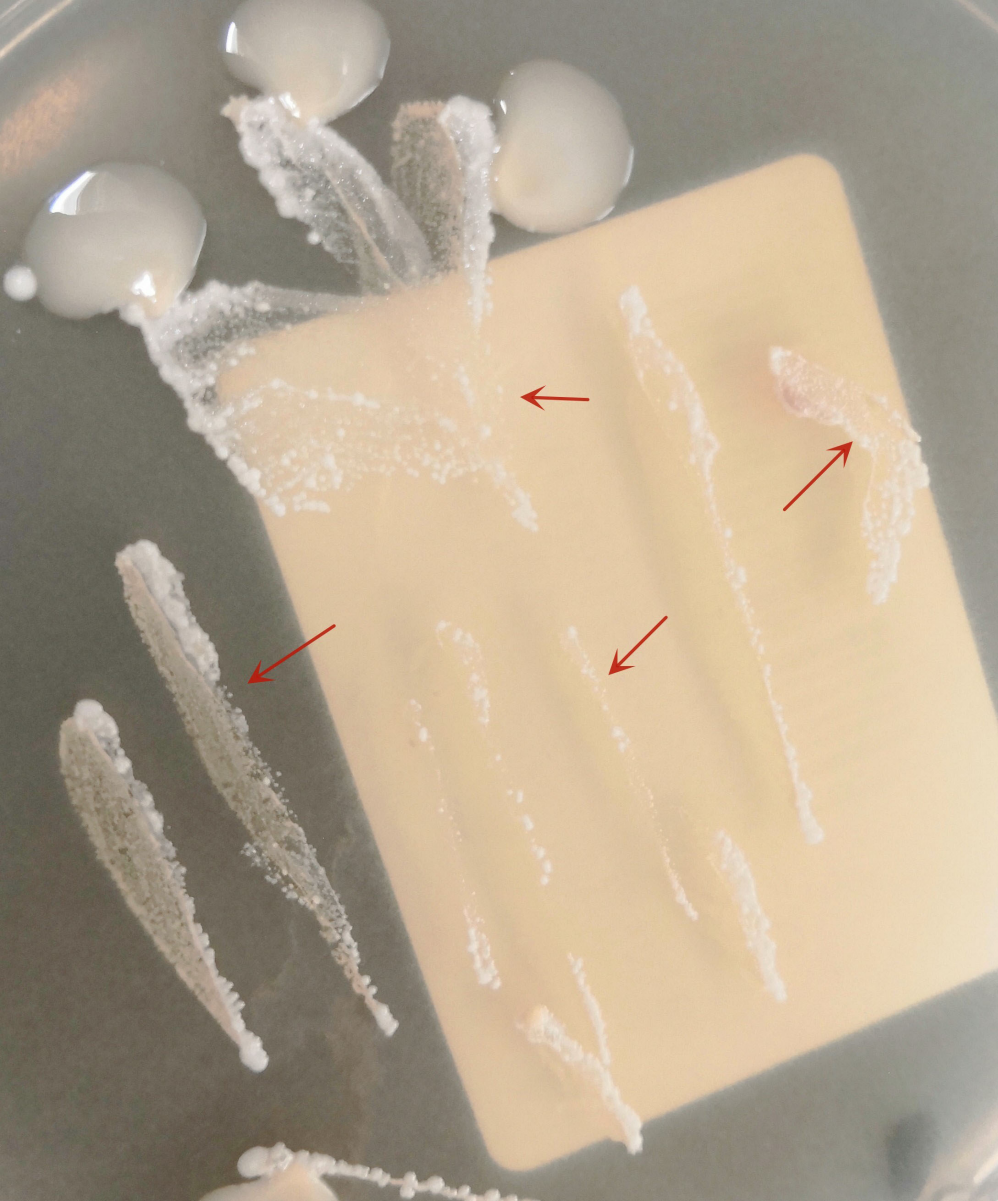
Primary colonies of *M. marinum* on PDA plate after 6 days of incubation at 25 °C (arrows).

**FIGURE 4:**
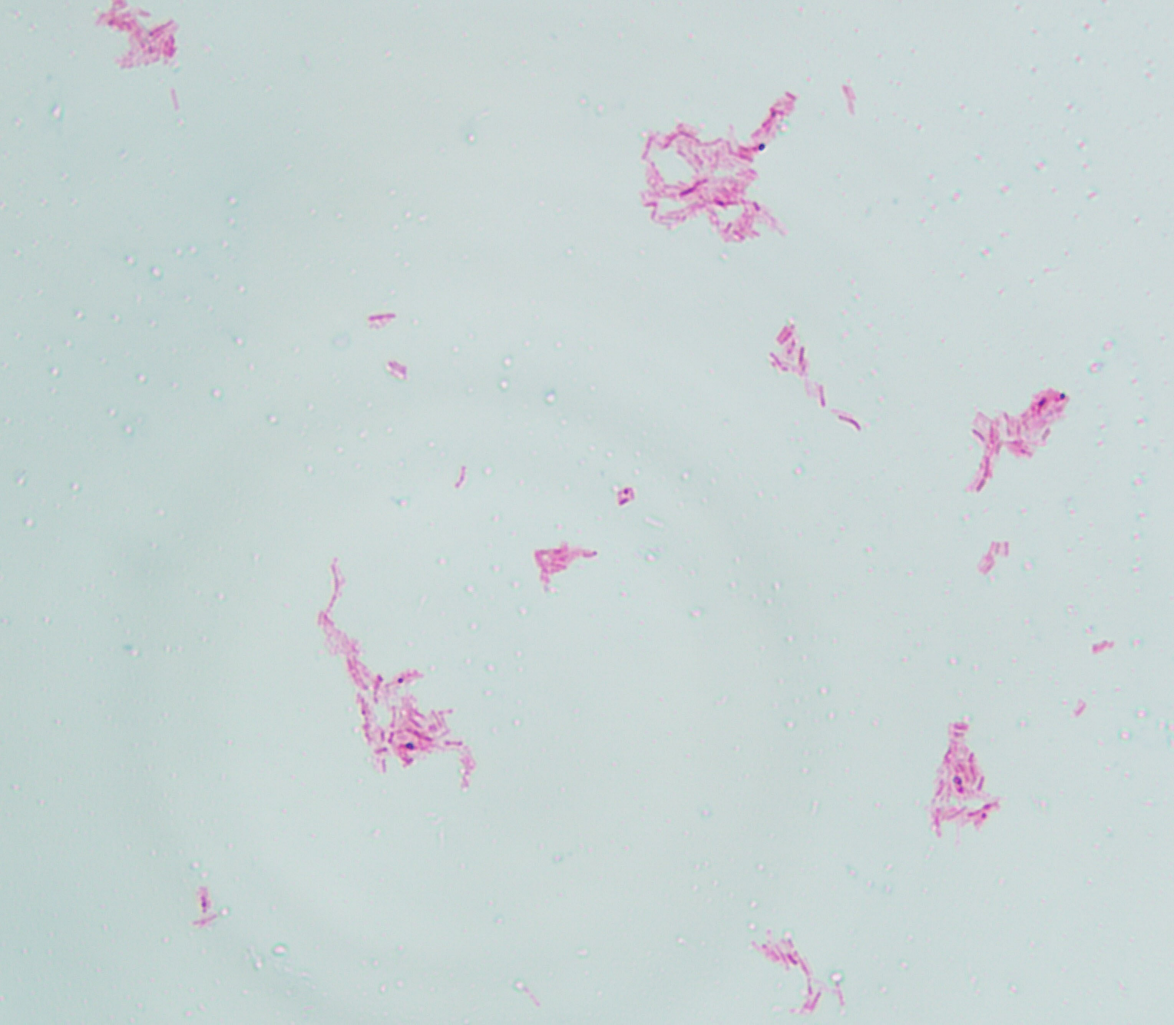
Acid-fast staining of colonies (×1000).

The pus material, obtained via needle puncture followed by extrusion, was inoculated onto chloramphenicol-containing Sabouraud Dextrose Agar (SDA) slants and PDA plates. To minimize contamination and moisture evaporation, the outer rim of each plate was sealed with laboratory tape. The inoculated media were then incubated at 25 °C. After 6 days of culture, PDA plates developed dense, white, raised granular colonies (Figure 3), whereas no growth was observed on SDA through day 14. Subcultured colonies were smeared onto slides for acid-fast staining, demonstrating numerous acid-fast bacilli (Figure 4). The isolate was identified as *M. marinum* by the matrix assisted laser desorption ionization time of flight mass spectrometry (MALDI-TOF MS) (Bruker Daltonik MALDI Biotyper) and the 16S ribosomal RNA (16S rRNA) sequencing (GenBank Accession No. PX128374). The mycological culture of nail debris samples confirmed the presence of *Trichophyton rubrum*. The patient was diagnosed with cutaneous NTM infection caused by *M. marinum* and treated with oral Doxycycline Hydrochloride Enteric-Coated Capsules (200 mg/d) for 6 months, resulting in complete resolution. No recurrence was reported during a 3-months telephone follow-up.

## 3 Discussion

Non-tuberculous mycobacterias are slender, non-motile, acid-fast bacilli (AFB) ubiquitously distributed in diverse environmental reservoirs. Runyon classified NTM into fast- and slow-growing groups, with the latter further subdivided based on pigment production under culture conditions (7). Almost all NTM species have been incriminated in cutaneous disease. The isolation and culture of NTM necessitate the use of specialized media, defined incubation temperatures, and extended incubation periods (8).

Historically, while classical media such as LJ and Middlebrook remain the standard, several studies have recommended the use of alternative, non-classical culture media for the cultivation of *Mycobacterium* spp (9–12). Broncano-Lavado et al. have shown that NTM Elite agar was effective for the recovery of NTM, especially for the RGM (13). Drancourt and Raoult demonstrated that *M. abscessus* grew equally well on blood agar as on Middlebrook 7H10 agar, a result consistent across all forty reference NTM isolates tested, with the exception of *M. ulcerans* (14). Some research efforts are primarily focused on enhancing and refining the detection methods for mycobacteria. Semi-automated blood culture systems using liquid media are recommended due to their ability to promote the growth of both *M. tuberculosis* complex (MTBC) and NTM. Nevertheless, despite these methodological advances, the high cost and limited accessibility of such techniques continue to pose significant barriers to their widespread implementation (15).

Potato dextrose agar is one of the most widely used media for the isolation and cultivation of various microbial strains (16), particularly in mycological laboratories. Its application traces back to the early 20th century (17). This medium supplies dextrose as a carbon source along with essential nutrients derived from potato extract, which creates a favorable environment for the growth of actinobacteria and fungi (17, 18). Furthermore, its formulation facilitates macroscopic colony observation, thereby supporting morphological characterization. PDA is composed of 1.5% agar and 2% glucose, with its nitrogen, phosphorus, vitamins, and trace nutrients primarily derived from a crude potato infusion (57.5 g of potatoes per liter of medium). Due to its high carbon-to-nutrient ratio, this medium supports not only robust growth of diverse fungal taxa but also promotes effective sporulation and the development of characteristic pigmentation. Currently, most mycologists use commercially available PDA formulations, which are typically prepared by mixing potato extract–often obtained through third-party extraction, drying, and powdering processes–with glucose and agar.

In addition to fungi, PDA is also suitable for isolating bacteria such as *Nocardia* spp (19). Our cases suggest that this medium may potentially be applied for the isolation of NTM, particularly *M. marinum*, though further validation of its suitability is warranted. It is worth noting that species identification still relies on MALDI-TOF MS or molecular sequencing. In summary, PDA medium is widely used in various medical institutions due to its low cost, easy accessibility, and ability to isolate a variety of microorganisms. In the context of RLS, the approach of using PDA for NTM isolation, along with acid-fast staining and clinical correlation, may represent a practical and viable diagnostic method for cutaneous NTM infections, warranting further comparative studies.

Cutaneous NTM infections typically arise following traumatic injury, surgical intervention, or cosmetic procedures. For trauma-associated cutaneous infections, it is generally recommended to collect specimens from the wound site within 48 h. However, this guidance typically pertains to major trauma, such as that from traffic accidents. In contrast, cutaneous NTM infections often result from inadvertent, minor trauma and follow an indolent clinical course. For such cases, tissue or aspirate specimens are considered optimal (20).

## Data Availability

The original contributions presented in this study are included in this article/supplementary material, further inquiries can be directed to the corresponding authors.
